# Two-factor synaptic plasticity enables memory consolidation during neuronal burst firing

**DOI:** 10.1093/pnasnexus/pgag213

**Published:** 2026-06-12

**Authors:** Kathleen Jacquerie, Danil Tyulmankov, Pierre Sacré, Guillaume Drion

**Affiliations:** Biology Department, Marder Lab, Brandeis University, 415 South Street, Waltham, MA 02453, USA; Department of Electrical Engineering and Computer Science, University of Liège, Allée de la Découverte 10, Liège 4000, Belgium; Center for Theoretical Neuroscience, Columbia University, 3227 Broadway, New York, NY 10027, USA; Viterbi School of Engineering, University of Southern California, 3670 Trousdale Parkway, Los Angeles, CA 90089, USA; Department of Electrical Engineering and Computer Science, University of Liège, Allée de la Découverte 10, Liège 4000, Belgium; Department of Electrical Engineering and Computer Science, University of Liège, Allée de la Découverte 10, Liège 4000, Belgium

**Keywords:** neuromodulation, eligibility trace, structural synaptic plasticity, neuromodulated synaptic plasticity

## Abstract

How can brain circuits remain plastic enough to encode new information while still stabilizing synaptic changes that support long-term memory? Many circuits switch between tonic spiking, which encodes external inputs, and burst firing, which is generated collectively; yet how these firing-state changes interact with synaptic plasticity to support consolidation remains unclear. Here, we ask whether burst epochs can provide a minimal, mechanistically interpretable route to stabilizing memories encoded during tonic firing. We introduce a two-factor synaptic plasticity rule in a conductance-based spiking network that switches robustly between tonic and burst regimes. The effective synaptic strength is expressed as the product of two factors: a primary, flexible factor updated by a Hebbian mechanism, and a secondary factor that captures stabilizing processes. The secondary factor is adjusted according to the rate of change of the primary factor. In a pattern recognition case study, the network encodes new inputs during tonic firing and undergoes burst epochs. This two-factor rule stabilizes previously learned patterns, integrates information across samples to support generalization, and improves robustness to noise. Ablation experiments show that these outcomes require a synergy between neural bursting activity and the two-factor plasticity rule: blocking secondary plasticity prevents stable retention, replacing bursts with quiescence leads to fading memories, and replacing bursts with additional tonic firing causes interference and noise sensitivity. Finally, a signal-to-noise ratio analysis across tonic-burst cycles identifies parameter regimes in which bursts either sharpen or weaken synaptic representations, consistent with consolidation or pruning, respectively.

Significance statement Brain circuits must learn from ongoing input while stabilizing synaptic change for consolidation. Many regions switch between tonic firing driven by inputs and collective burst firing, yet how burst firing supports memory consolidation remains unclear. We introduce a minimal two-factor rule in a conductance-based network that switches between tonic and burst regimes: a flexible primary factor encodes activity, while a secondary factor stabilizes tonic-driven changes during bursts. In a memory task, this mechanism consolidates stored patterns and improves robustness to noise. Replacing bursts with quiescence yields fading memories, and replacing bursts with additional tonic episodes encoding external stimulus produces interference. Together, these results suggest that brief burst epochs can provide a simple, mechanistically interpretable substrate for memory consolidation.

## Introduction

Neurons adjust their connectivity through synaptic plasticity—a process in which correlated activity engages intracellular signaling cascades that modify synaptic efficacy and, often, synapse structure. Calcium influx into the postsynaptic compartment can trigger phosphorylation and trafficking of postsynaptic receptors, and may be accompanied by structural remodeling such as changes in dendritic spine volume (1–3). These mechanisms provide a substrate for learning and memory, yet raise a long-standing computational challenge: how can synapses remain sufficiently plastic to encode new information while also maintaining stability and homeostasis over long timescales (4–8).

Furthermore, some brain circuits naturally fluctuate between asynchronous tonic firing and collective bursting depending on behavioral context and neuromodulatory state (9). During tonic firing, neurons emit spikes that primarily track ongoing external input. Burst firing consists of brief periods of high-rate spiking activity separated by silent periods, and can arise from intrinsic and network mechanisms, including cycles of activation and inactivation of voltage-gated conductances such as T-type calcium channels (10, 11). When bursting becomes synchronized at the population level, it produces large-amplitude, low-frequency components in local field potentials. Although bursts are classically associated with sleep and drowsiness, burst-like events can also occur during wakefulness, including transient sleep-like activity and local offline periods in cortex (12–14). These observations support the view that wakefulness is not a uniform dynamical state, but can include transient, spatially localized episodes with sleep-like population dynamics that are less responsive to external input.

Despite the prevalence of state switching, it remains unclear how tonic and burst regimes interact with synaptic plasticity to support memory consolidation. Addressing this question is difficult for three reasons. First, much of the plasticity theoretical literature explores tonic spiking and bursting in isolation, whereas comparatively fewer models examine learning in networks that alternate between these regimes (15–17). Second, consolidation is often modeled at the systems level through multihour sleep and multiregion dynamics, using mechanisms such as replay, renormalization, or pruning—approaches that can be powerful but add substantial modeling complexity (18–20). Third, biological models that explicitly track postsynaptic signaling cascades and structural changes can become high-dimensional and difficult to scale in neuronal networks (21, 22).

To address this gap, we ask whether burst epochs can provide a minimal, mechanistically interpretable route to consolidation in a network that alternates between tonic firing and burst activity. Building on our conductance-based model that robustly switches between tonic and burst regimes (23), we introduce a two-factor synaptic plasticity rule in which the effective synaptic strength is expressed as the product of two factors: a primary, flexible factor governed by a Hebbian plasticity rule, and a secondary factor capturing stabilizing processes. The secondary factor is adjusted according to the rate of change of the primary factor. This coarse-grained formulation lumps multiple subsynaptic components into a compact description (24). In this sense, it serves as a compact proxy for processes that support synaptic stability without explicitly modeling the full complexity of signaling pathways, making it possible to investigate consolidation in a network model that remains both tractable and mechanistically interpretable.

We evaluate the rule in a pattern recognition task using images of handwritten digits, in which the network encodes new digits during tonic firing and consolidates them during subsequent burst epochs. We quantify performance using two complementary measures: preservation of learned synaptic structure across tonic-burst cycles, and classification accuracy on training and unseen test samples. Together, these readouts capture the degree of consolidation and its resultant effect on generalization performance. The two-factor rule yields three key functional outcomes: it stabilizes previously learned patterns (consolidation), is able to make correct predictions on new inputs (generalization), and improves robustness to noise. Ablation experiments show that these properties do not arise from bursts or tonic firing alone: blocking the plasticity in the secondary factor leads to loss of stable retention; replacing bursting neurons with quiescent ones causes memories to fade; and replacing burst firing with additional tonic firing, associated with encoding of new digits, produces interference and noise sensitivity. Finally, by sweeping key model parameters and quantifying selectivity with a signal-to-noise ratio (SNR) across successive tonic–burst cycles, we show that burst epochs can either sharpen representations or weaken them.

## Results

### Modeling robust tonic and burst firing in a biophysical network model

**Figure 1:**
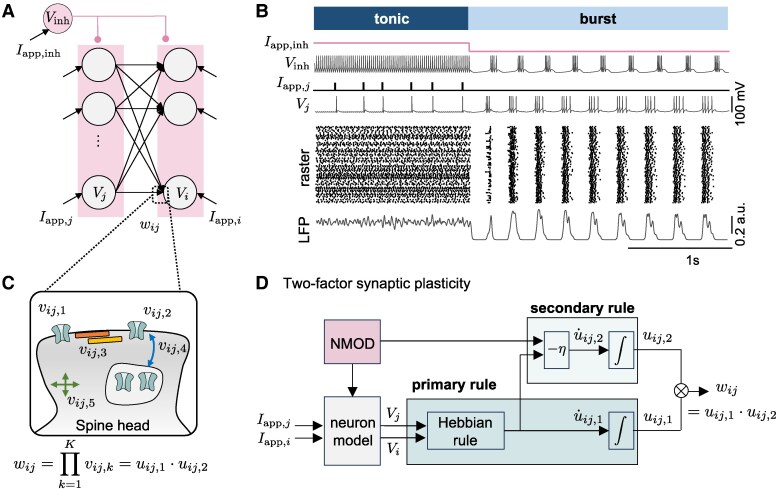
A biophysical network model of tonic-burst regimes with two-factor synaptic plasticity. A) Network architecture: one inhibitory neuron ($V_{\mathrm{inh}}$) projects to all excitatory neurons, which are connected feedforward from *N* presynaptic neurons ($V_{j}$) to *M* postsynaptic neurons ($V_{i}$). An external current to the inhibitory neuron ($I_{\mathrm{inh}}$) controls the network state; each excitatory neuron receives an independent external current mimicking input (from Ref. (25)). B) Hyperpolarizing $I_{\mathrm{inh}}$ switches the network from tonic firing encoding external inputs to collective burst firing, shown through membrane potentials, raster plot, and LFP traces (A and B adapted from Ref. (25)). Dark blue box = tonic epoch (on the left); light blue box = burst epoch (on the right). C) Schematic of the synapse model. The total synaptic weight $w_{ij}$ is the product of many subsynaptic components in the spine head; for example representing the efficacy and the number of the postsynaptic receptors ($v_{ij,1}$, $v_{ij,2}$), the scaffolding proteins ($v_{ij,3}$), the insertion of new receptors via exocytosis ($v_{ij,4})$, or structural change ($v_{ij,5}$) among other. We lump these subsynaptic components into two factors; $u_{ij,1}$ and $u_{ij,2}$ (inspired by Ref. (24)). D) Two-factor synaptic plasticity illustrated in the state diagram. The primary plasticity on $u_{ij,1}$ operates during both tonic and burst states. It follows a Hebbian rule driven by pre- and postsynaptic activity. The secondary plasticity on $u_{ij,2}$ is active only during burst firing. It couples the two factors through the rate of change of the primary factor via a coupling gain *η*. Neuromodulation (NMOD) can switch the firing pattern of the neurons, turn on-and-off or tune *η*.

To model a brain circuit that can reliably fire in both tonic and burst regimes, we use a conductance-based spiking neural network introduced in our previous work (25) (see also “Methods”). The architecture consists of *N* presynaptic excitatory neurons with membrane potentials $V_{i}$ projecting to *M* postsynaptic excitatory neurons with membrane potentials $V_{j}$. All excitatory neurons receive inhibition from a single pacemaking inhibitory neuron (Fig. 1A) which regulates the network state, mimicking the role of neuromodulators such as acetylcholine, dopamine, serotonin, or histamine—known to control brain state transitions (23, 26–29). This architecture can be interpreted as a stereotyped subnetwork that encodes memory, while receiving a coarse global inhibition signal. We do not aim to reproduce the full inhibitory-excitatory microcircuitry of cortex or hippocampus; rather, the single inhibitory neuron provides a simple control knob that allows us to separate tonic firing encoding external inputs from offline periods in bursting regime. In this reduced setting, switching is implemented by modulating the applied current to the inhibitory neuron, $I_{\mathrm{app},\mathrm{inh}}$, as an abstract proxy for inhibitory and neuromodulatory drive.

During *tonic firing encoding external inputs*, a depolarizing current $I_{\mathrm{app},\mathrm{inh}}$ applied to the inhibitory pacemaker neuron induces regular tonic spiking and sets the excitatory population resting membrane potential. In this regime, excitatory neurons fire primarily in response to their applied input currents ($I_{\mathrm{app},j}$, $I_{\mathrm{app},i}$; see “Methods” section).

To switch the network into *collective burst firing*, we apply a hyperpolarizing $I_{\mathrm{app},\mathrm{inh}}$ to the inhibitory neuron. In this regime, excitatory neurons generate synchronized clusters of spikes separated by silent periods. Importantly, bursting is generated endogenously by intrinsic membrane dynamics based on intrinsic conductances such as T-type calcium channels and inhibition: once the network is in the burst regime, we silence external inputs in $I_{\mathrm{app},j}$ and $I_{\mathrm{app},i}$ and retain only a small noisy baseline current, which does not by itself elicit spiking. Thus, the timing and occurrence of bursts are not imposed by external patterned input, but emerge from intrinsic neuronal properties, including deinactivation of T-type calcium channels under hyperpolarization (13, 23, 25, 30).

At the population level, these two regimes produce distinct local field potential (LFP) signatures: fast, low-amplitude oscillations during tonic activity and slow, high-amplitude oscillations during burst firing (Fig. 1A and B, see “Methods” section). This switching mechanism provides a biologically plausible framework for studying how synaptic plasticity operates across alternating neural firing regimes.

### The coupled two-factor synaptic plasticity rule

Two neurons interact through synapses whose functional strength reflects the combined state of multiple subsynaptic processes, including presynaptic release properties (eg vesicle availability and release probability), postsynaptic receptor number and efficacy, and structural organization of the synapse (3, 24, 31). Synaptic plasticity recruits biochemical and structural processes that act on these subsynaptic quantities by modifying their concentrations, configurations, and interactions over multiple timescales (24). We schematize this idea in Fig. 1C: correlated activity can engage diverse pathways affecting, for example, receptor phosphorylation ($v_{ij,1}$), receptor trafficking ($v_{ij,2}$), scaffold organization ($v_{ij,3}$), exocytosis ($v_{ij,4}$), and spine morphology ($v_{ij,5}$).

Inspired by prior work (24, 32, 33), we represent the total synaptic efficacy more generally as the product of *K* subsynaptic components,


$$w_{ij}=\prod_{k=1}^{K}v_{ij,k}.$$

However, explicitly modeling the kinetics of each $v_{ij,k}$ would require a high-dimensional biochemical description and detailed knowledge of pathways that remain incomplete. Moreover, our goal is to understand how plasticity interacts with tonic and burst regimes under neuromodulatory drive, which motivates a reduced description that remains tractable at the network level. Therefore, unlike the prior work, we abstract these subsynaptic components into two simple phenomenological factors:


(1) $$w_{ij}=u_{ij,1}\cdot u_{ij,2},$$

where $u_{ij,1}$ is a flexible factor that captures rapid synaptic change due to correlated neuronal activity and $u_{ij,2}$ captures stabilizing processes recruited during burst epochs.

The primary factor follows a Hebbian plasticity rule that is active during both tonic and burst regimes (Fig. 1D). We write its dynamics as


$${\dot{u}}_{ij,1}=f(V_{\;j},V_{i}),$$

and use a calcium-based rule as a concrete instance (34, 35) (see “Methods” section). In previous work, we showed that burst firing drives a reorganization of synaptic weights across multiple Hebbian formulations (including spike-time dependent plasticity, STDP, and triplet rules) (25, 36).

We assume that the secondary factor is only engaged during burst firing. Burst epochs are accompanied by large changes in network state and neuromodulatory tone, which can recruit signaling pathways distinct from those dominating during tonic firing. Rather than using a detailed biochemical model of pathways that remain incompletely characterized, we introduce a phenomenological coupling in which the rate of change of the secondary factor tracks the rate of change of the primary factor:


(2) $${\dot{u}}_{ij,2}=-\eta_{ij}(X){\dot{u}}_{ij,1},$$

where $\eta_{ij}(X)\geq 0$ is a coupling gain. In the most general case, $\eta_{ij}$ can depend on a vector of circuit or neuromodulatory variables *X*, allowing it to differ across regimes. Here, we take $X=I_{\mathrm{app},\mathrm{inh}}$, the signal that switches the network between tonic and burst regimes, and assume a uniform gain across synapses:


$$\eta_{ij}(I_{\mathrm{app},\mathrm{inh}})=\eta \Theta \left(I_{\mathrm{app},\mathrm{inh}}-\theta_{\mathrm{thres}}\right),$$

so that the secondary rule is engaged only during bursts (ie when $I_{\mathrm{app},\mathrm{inh}}$ exceeds a threshold) and ${\dot{u}}_{ij,2}=0$ during tonic states. We denote the global coupling gain by *η*. Increasing *η* produces larger burst-driven changes in $u_{ij,2}$, reflecting tighter coupling between the two factors (Fig. 1D). The effects of varying *η* are analyzed in “Coupling gain and initial state affect consolidation dynamics during burst firing,” section and the possibility of heterogeneous, synapse-specific modulation is mentioned in the discussion.

### Synaptic plasticity across tonic, burst, and quiescent network firing regimes

**Figure 2:**
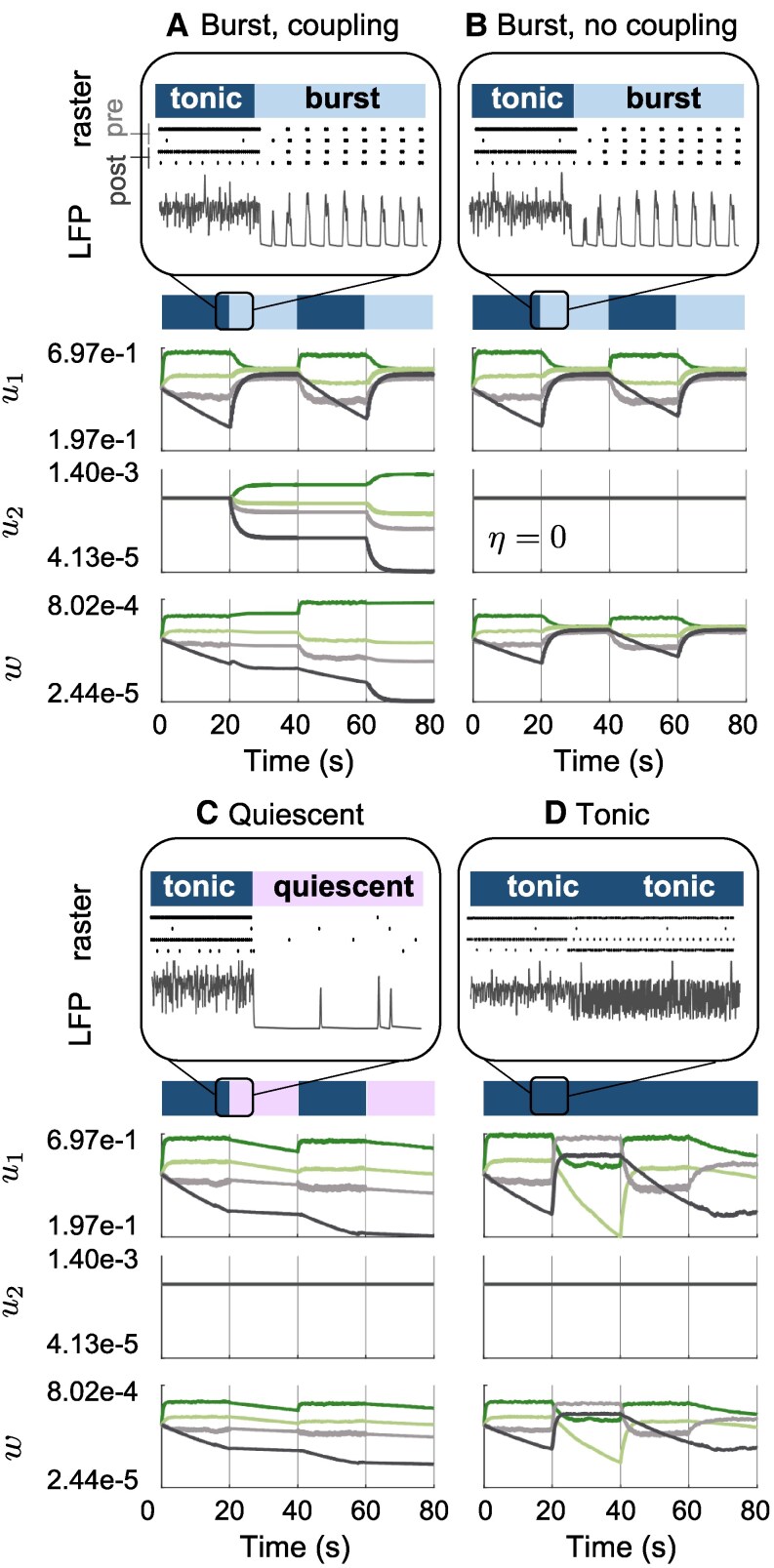
A–D) Evolution of primary factor ($u_{1}$), secondary factor ($u_{2}$), and total (*w*) weights in a two-by-two neuron circuit with varying correlation levels. Each color tracks one synapse. Dark blue = tonic epoch; light blue = burst epoch; lavender = quiescent. A) Burst with secondary plasticity (η=1/500) (“Burst, coupling” model): when $u_{1}$ resets during burst firing, the external inputs encoded during tonic firing are transferred to $u_{2}$, so the total weight consolidates across bursts. B) Burst without the secondary plasticity (η=0) (“Burst, no coupling” model): only the primary plasticity acts; burst firing drives weights toward the burst-induced attractor. C) Quiescent neurons replaces bursting neurons (“Quiescent” model); the weights decay slowly (η=0). D) Tonic firing replaces burst firing (“Tonic” model); the weights encode new memories (η=0).

We first examine how the proposed two-factor plasticity rule influences memory consolidation in a simple circuit with two presynaptic and two postsynaptic neurons. The presynaptic neurons fire at distinct tonic frequencies, producing heterogeneous synaptic trajectories during tonic activity (see “Methods” section for details). In Fig. 2, synapses driven by strongly correlated activity potentiate (green), whereas synapses driven by weakly correlated activity depress (gray), leading to a separation of $u_{1}$ values. We interpret $u_{1}$ as a labile, synaptic tag-like component that reflects recent activity through fast expression mechanisms (eg changes in receptor efficacy, phosphorylation, or rapidly recruited pathways).

When the network enters bursting, the primary factors converge toward the same narrow range of values. In our previous work (25), we termed this phenomenon as the *burst-induced attractor*. Intuitively, collective bursting synchronizes plasticity-driving signals across synapses, homogenizing their dynamics and forcing convergence toward shared fixed points. As a result, $u_{1}$ is pulled toward a narrow region (attractor), effectively acting as a “reset.” These dynamics motivate coupling the primary and secondary factors.

In our framework, burst epochs correspond to a distinct network state in which the neuromodulatory and intracellular signaling context may differ from that of tonic encoding. If bursts only reset $u_{1}$, previously encoded differences would be erased. The role of the secondary factor $u_{2}$ is therefore to transfer and stabilize this information during the burst-driven convergence of $u_{1}$ (37, 38).

Mechanistically, consolidation arises because during bursts, the secondary factor $u_{2}$ tracks the rate of change of the primary factor $u_{1}$. Thus, as $u_{1}$ converges to the attractor, $u_{2}$ changes in a compensatory direction, transferring information encoded during tonic firing into a more stable variable. Once $u_{1}$ reaches the attractor and its derivative vanishes, $u_{2}$ stabilizes as well. Consequently, the effective weight $w=u_{1}\cdot u_{2}$ preserves the relative differences acquired during tonic firing even though $u_{1}$ is reset by burst dynamics. Illustrated in Fig. 2A (“Burst, coupling”), this model of tonic and burst firing combined with the coupled two-factor rule provides an interpretable route to consolidation without explicitly modeling the full biochemical cascade.

To assess the role of the two-factor plasticity, we disable the coupling between the primary and secondary factor by setting η=0, blocking the secondary plasticity rule, leaving only the primary Hebbian mechanism (Fig. 2B). We define this model as “Burst, no coupling” for the rest of the work. In this case, tonic firing increases the primary factor, but the subsequent burst drives all $u_{1}$ values toward the attractor. Without secondary plasticity, this convergence erases previously encoded information, leaving only transient traces. Biologically, this resembles blocking cascade signaling pathways required for long-lasting plasticity (eg preventing structural change or receptor insertion) (39, 40).

A common modeling approach is to alternate learning phases with offline phases treated as periods of inactivity (41). We therefore ask whether burst firing could be replaced by such a quiescent regime. In the “Quiescent” model (Fig. 2C), synaptic weights change during tonic firing, but the subsequent quiescent phase provides no mechanism for stabilization. As a result, the total weights decay slowly and retain only short-lived information. Because burst firing is absent in this condition, the secondary plasticity rule is never engaged.

Finally, we test the effect of replacing burst episodes with additional tonic episodes. In the tonic only model (Fig. 2D, η=0), each burst period is substituted by another tonic phase in which new inputs are presented. Instead of consolidating prior information, these additional tonic episodes overwrite previously encoded patterns, preventing memory stabilization. This phenomenon is commonly referred to as catastrophic forgetting in the literature (42).

Together, these comparisons show that burst firing *combined* with the coupled two-factor plasticity is uniquely suited to preserve and stabilize memories: bursting alone triggers the burst-induced attractor, quiescence allows only transient storage, and continuous tonic firing induces interference. In contrast, when using the two-factor rule, burst epochs actively consolidate information encoded during tonic firing.

### Two-factor synaptic plasticity promotes pattern consolidation during burst firing

We next test whether the proposed two-factor synapse model supports memory consolidation in a pattern recognition task similar to previous work (41, 43). The network is trained on the MNIST dataset of handwritten digits (44), using downsampled 22×22 images for computational efficiency. Each of the N=484 presynaptic neurons encodes the intensity of a single pixel by its firing rate, while M=10 postsynaptic neurons represent the ten digit classes in a “one-hot” encoding.

**Figure 3:**
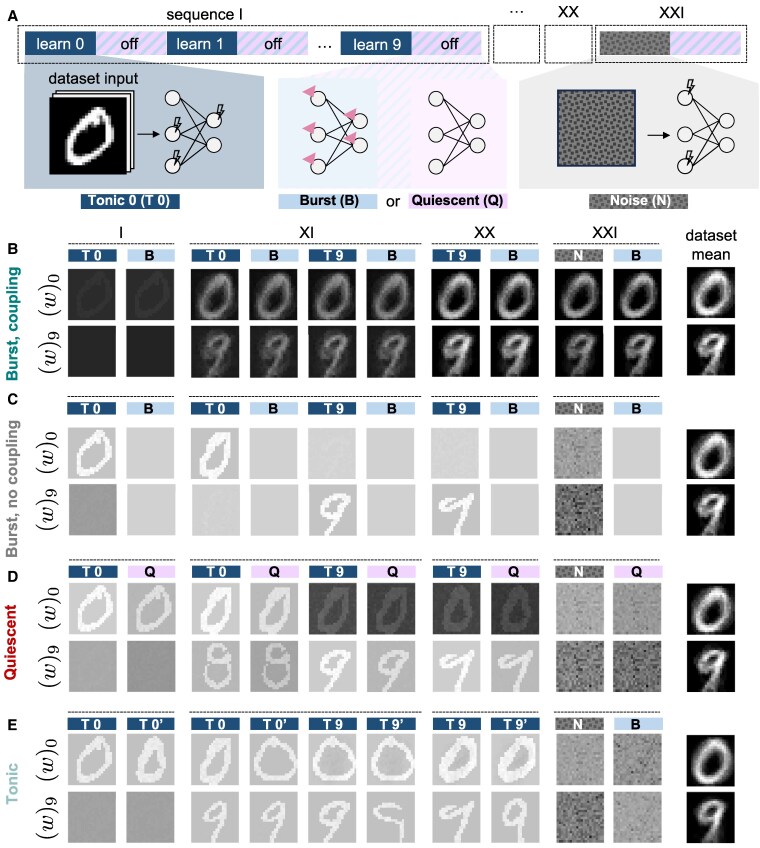
Memory consolidation through burst firing and two-factor plasticity. A) Schematic of the experimental protocol. The network sequentially encodes digits (0–9) during tonic firing periods (T) where neurons receive external inputs (lightening), interleaved with offline periods (hatched lavender, blue), which can be modeled either as burst firing (B) or quiescence (Q). It consists of 20 sequences (I–XX). In the final sequence, random noise is presented (N) on the last sequence (XXI). B–E) The weight matrices are shown for the output neurons associated with digits 0 and 9 at the transition between epochs within one sequence (noted $(w{)}_{0}$ and $(w{)}_{9}$). The gray color scale is normalized between the minimum and maximum weight of the simulation. The last column is the mean of the 20 dataset images for the digits 0 and 9 presented during the 20 sequences. B) Burst with secondary plasticity (η=1/400, “Burst, coupling” model): secondary weights preserve information learned during tonic firing, enabling the total weight (*w*) to retain and consolidate memories across sequences. Dark blue box containing T = tonic epoch; light blue box containing B = burst epoch. C) Burst without secondary plasticity (η=0, “Burst, no coupling” model): During burst firing, the weights return to baseline, leading to loss of previously encoded patterns. D) “Quiescent” model (η=0): quiescent neurons replaces bursting neurons; weights decay gradually and memories are not consolidated. Lavender box containing Q = quiescent epoch. E) “Tonic” model (η=0): offline periods are replaced by additional tonic firing periods for sequences I to XX (T *i*’). The network only encodes the current digit, forgets previous learning, and is fragile to noise. The last column is the mean of the 40 dataset images presented during the 40 tonic firing states.

Training consists of 21 sequences (Fig. 3A, Roman numerals I–XXI), with each sequence composed of 10 blocks. For sequences I through XX, in block *i* (for i=0,1,…,9), a randomly sampled image of digit *i* is presented to the network in a tonic firing phase (Fig. 3A, dark blue, T), followed by either collective bursting (Fig. 3A, light blue, B), quiescence (Fig. 3A, lavender, Q), or an additional tonic firing period (T’), as in the previous section. The final sequence XXI tests robustness by presenting noise input (N). For more details about the implementation, see “Methods” section.

To visualize learning progress, we monitor the set of synaptic weights associated with each postsynaptic neuron, corresponding to a unique digit. The set of effective synaptic weights for digit *i* is defined as the element-wise product of primary and secondary factors afferent onto neuron *i*, in other words $w_{i}=u_{i\;:\;,1}⊙u_{i\;:\;,2}$. For visualization, this set of weights is then reshaped into a 22×22 matrix to recover the pixel structure. Examples for digits 0 and 9 are shown in Fig. 3B–E (all digits shown in Fig. S1).

The results highlight the synergy between bursting and two-factor plasticity. In the “Burst, coupling” model, information is preserved: the secondary factor transfers learning from tonic periods into a stable component, enabling the total weights to retain clear digit representations across sequences (Fig. 3B, η=1/400). This demonstrates a form of memory consolidation that does not rely on explicit replay of previously learned patterns, which is a common mechanism in many consolidation frameworks (43, 45). In addition, the network forms an integrated representation across different digit samples. This is evident in the weight matrices, which do not simply reflect the most recent digit, but instead combine features from samples presented during earlier tonic firing states.

To assess the role of the two-factor plasticity, we disable the coupling by setting η=0, leaving only the primary mechanism (“Burst, no coupling” model). Burst firing erases previously learned patterns by driving weights toward the burst-induced attractor (Fig. 3C, shown by the gray pixel structure). In comparison, we replace burst firing by a quiescent period, used in computational models to reproduce inactivity in a network. The “Quiescent model” only encodes the current digit, then leads to gradual weight decay and poor retention (Fig. 3D). Additional learning can be seen as an appealing strategy for learning more information instead of alternating between tonic and burst firing. However, we observe that the “Tonic model” overwrites previously stored digits with each new input, preventing consolidation and leaving the network fragile to noise (Fig. 3E). We further discuss the effect of modifying the primary plasticity rule with another calcium-based model in Fig. S3. We observe that different types of learning occur for overlapping pixels.

Together, these ablation experiments show that consolidation emerges only when burst firing is combined with the two-factor rule.

### The two-factor synaptic plasticity rule supports memory retention and generalization

**Figure 4:**
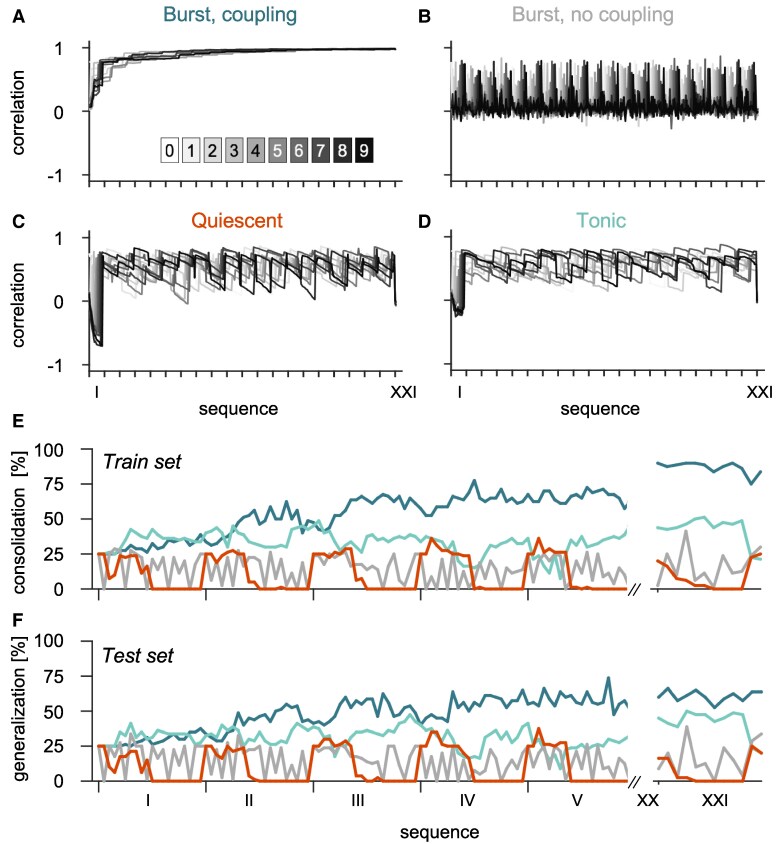
The two-factor synaptic plasticity rule supports memory retention and generalization. A–D) Correlation between the synaptic weight matrices and the average digit images from the training dataset across learning sequences (I–XXI). Each trace corresponds to one digit class (0–9), shown in grayscale from light (digit 0) to dark (digit 9). A) Burst with secondary plasticity: weights progressively align with digit samples, showing stable correlation. B) Burst without secondary plasticity: weights reset at each burst, erasing previously stored information. C) Quiescent model: quiescence replaces bursts; weights drift and correlations gradually decay. D) Tonic model: performance fluctuates, with partial correlations but no consolidation across sequences. E and F) Accuracy percentage during digit classification for sequences I–V, XX, and XXI, measured on (E) the training set (80 samples) defined as consolidation, and F) an unseen test set (80 samples), defined as generalization. Burst with two-factor plasticity (dark teal, top final trace) is generalizing the dataset. Tonic model (light teal, second trace) performs moderately but fails to generalize across sequences. Burst without two-factor plasticity (gray, showing sawtooth pattern) and Quiescent model (red) fail to maintain learned patterns and show poor generalization.

To quantify the learning process, we compute the correlation between the weights encoding each digit and its template, the mean image of the corresponding digit class in the training dataset (the mean of the 20 training images for digits 0 and 9 is shown in the last column of Fig. 3B–E). A high correlation indicates that the synaptic weight pattern closely resembles the template of that digit, meaning that the network retains the dataset mean of samples; a low or negative correlation indicates that the representation has been lost or overwritten. This measure provides a direct and interpretable proxy for memory retention between the different network states. The “Burst, coupling” model preserves high digit correlations across sequences despite noise (Fig. 4A). In contrast, removing the coupling rule, replacing burst firing by quiescent periods or stonic periods yields only transient correlations (Fig. 4B–D). Thus, bursting helps to maintain learned representations.

Although correlation captures how closely synaptic weights resemble digit templates, it does not directly quantify how well the network *uses* these weights to recognize inputs. We therefore measure *classification accuracy* at the end of each block. In our protocol, accuracy on previously learned digit classes provides an operational measure of *consolidation*: if accuracy remains high after subsequent learning blocks and after the intervening offline phase (burst or quiescence), then earlier representations have been retained rather than overwritten. To assess *generalization*, we repeat the same evaluation on a held-out dataset: accuracy on unseen samples of the same digit classes reports how well learned synaptic structure transfers beyond the specific training examples.

The accuracy is obtained by testing the network on MNIST digits used *during* the training protocol under the four models. At the end of each block (ie at the end of each firing regime epoch), the network is presented with 20 samples of each digit from 0 to 3. These samples are either drawn from the training set (used in learning, Fig. 3A) or from an unseen test set. This evaluation does not involve synaptic plasticity, allowing us to measure recognition using the learned weights. Predictions are assigned to the output neuron with the highest spike count, and the accuracy is calculated as the fraction of correct predictions. To ensure comparability, models without secondary plasticity are normalized by the maximum weight value from the “Burst, coupling” model (see “Methods”; unnormalized results in Fig. S2).

On the training set, the “Burst, coupling” model shows steadily increasing accuracy across sequences and clearly outperforms the other models (Fig. 4E), indicating robust retention of previously learned classes despite continued learning. The “Burst, no coupling” model shows a sawtooth pattern, with accuracy dropping after each burst as weights are pulled toward the burst-induced attractor. The “Quiescent” model learns digits sequentially but fails to maintain them, whereas the “Tonic” model exhibits interference as each new digit overwrites earlier ones. The accuracy stays below 50%. By the final noise sequence, only the “Burst, coupling” model maintains robust performance.

On the test set, the “Burst, coupling” model also achieves the highest accuracy (Fig. 4F), demonstrating improved generalization to unseen samples. Although absolute accuracy is lower than optimized machine-learning systems, our aim is not to maximize MNIST performance but to use accuracy as a functional readout of retention and transfer in a biophysically grounded network operating across tonic and burst regimes.

### Coupling gain and initial state affect consolidation dynamics during burst firing

So far we have presented results using fixed values of the coupling gain, η=1/500 in the small circuit (Fig. 2) and η=1/400 for MNIST (Fig. 3), and initial secondary factor, $u_{2}(0)=0.001$. In practice, we also observe that consolidation dynamics vary depending on these values. Here, we investigate these effects more systematically in a simplified network. Following the computational protocol of (46), we assess the information stored in the network across successive states by computing the SNR.

**Figure 5:**
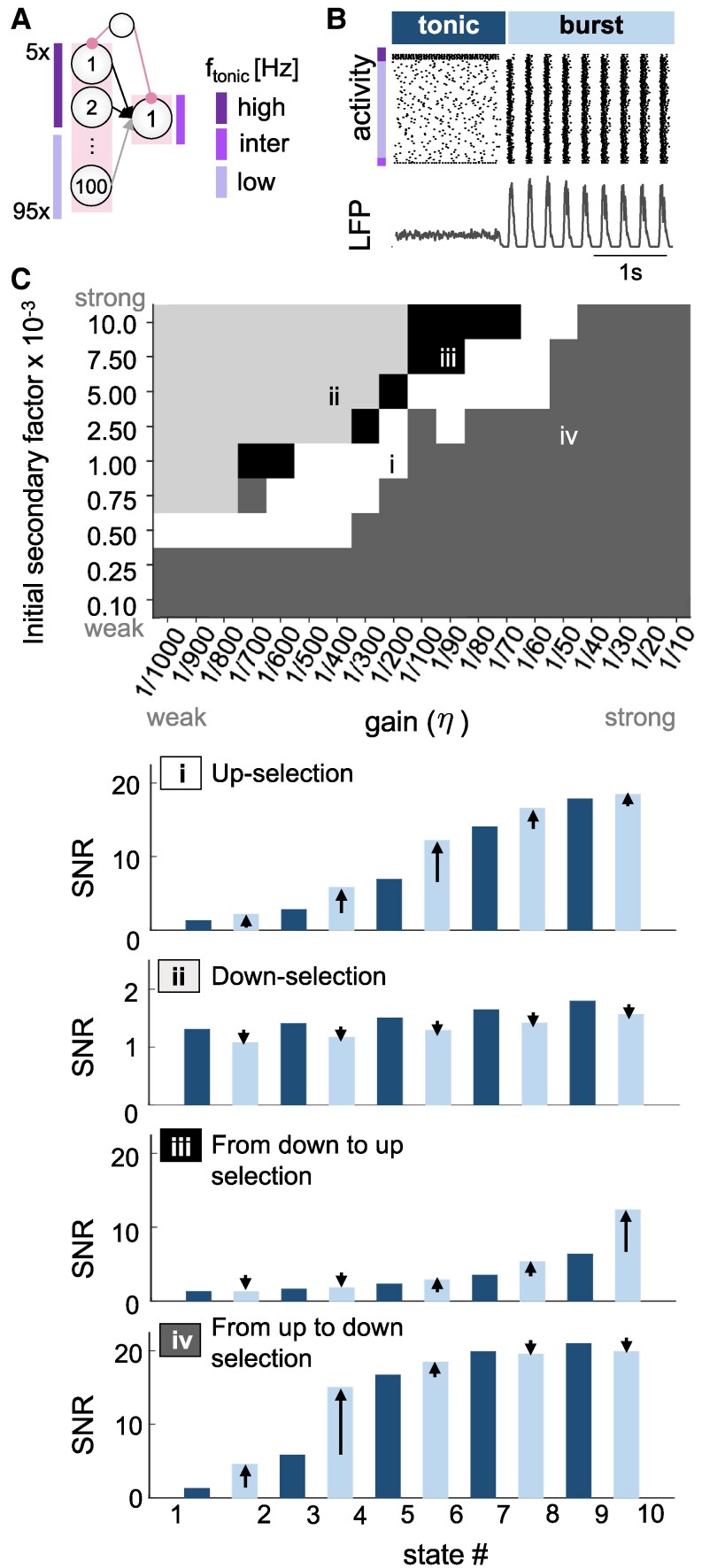
Coupling gain and initial state control synaptic selection during burst firing. A) The effect of the coupling gain *η* is studied in a network with one inhibitory neuron projecting to 100 presynaptic neurons connected to a single postsynaptic neuron. The network undergoes 10 cycles of 20 s, alternating between tonic and burst firing. Presynaptic neurons fire at different tonic frequencies (high, intermediate, or low; shades of purple). B) Raster plot of excitatory activity and LFP during the first switch from tonic to burst firing. C) Evolution of the SNR across successive tonic and burst states under different initial secondary factor $u_{2}(0)$ and coupling gain (*η*). Ci) SNR increases during burst firing (up-selection; white, η=1/200, $u_{2}(t=0)=0.001$). Cii) SNR decreases during burst firing (down-selection; light gray, η=1/400, $u_{2}(t=0)=0.005$). Ciii) Transition from down- to up-selection during the simulation (black, η=1/90, $u_{2}(t=0)=0.0075$). Civ) Transition from up- to down-selection (dark gray, η=1/50, $u_{2}(t=0)=0.0025$).

We consider a network of 100 presynaptic and 1 postsynaptic neuron (N=100, M=1, Fig. 5A), which undergoes 10 alternating states of tonic and burst firing. During each state of tonic firing, 5 randomly selected presynaptic neurons are stimulated by a high-frequency pulse train ranging from 73 to 76 Hz (Fig. 5B, dark purple), while the remaining 95 neurons receive stimulation from a low-frequency pulse train ranging from 0.1 to 5 Hz (Fig. 5B, lilac). The output neuron is stimulated by a pulse train at an intermediate frequency, fixed at 25 Hz (purple). Synapses undergo the primary and secondary plasticity as introduced in “The coupled two-factor synaptic plasticity rule” section. During tonic firing, the five neurons firing at a high frequency potentiate their connection with the output neuron, while the rest tend to depress, as dictated by the calcium-based rule on the primary factor.

We define the SNR as the maximum total weight divided by the mean of all total weights (Eq. 4) (46). Overall, SNR tends to increase over the course of the simulation (Fig. 5Ci–Civ). However, for varying values of *η* and $u_{ij,2}(0)$, four different microscopic trends emerge across successive tonic and burst firing states (Fig. 5C, top). In the first, which we term *up-selection*, the SNR after the burst state is higher than after the preceding tonic state (Fig. 5Ci). In other words, burst firing increases synaptic contrast without replay, by selectively strengthening the most informative weights relative to weaker ones. Conversely, during *down-selection*, the SNR decreases during burst firing (Fig. 5Cii), indicating a reduction in synaptic contrast (eg weakening of strong weights and/or reduced separation between strong and weak weights). Both mechanisms can coexist: the SNR can initially decrease during burst firing and then increase, in a *transition from down- to up-selection* (Fig. 5Ciii), as well as the reverse, starting with an increase and then a decrease, in a *transition from up- to down-selection* (Fig. 5Civ).

The network can navigate within these four microscopic trends by varying the value of the gain *η*, tuned through the action of neuromodulators, which can influence cascade signaling pathways on a global scale (47–52) and also the initial secondary factor. For example, dopamine or acetylcholine can affect learning on the whole network (51). Furthermore, these pathways can be locally modulated for specific synapses through synaptic-tagging and capture mechanisms (STC) (53–55). According to this theory, synapses tagged after correlated pre- and postsynaptic activity experience enhanced protein synthesis, leading to synapse consolidation.

The effect of the gain, however, varies depending on the initial secondary factor connectivity $u_{ij,2}(0)$. For low values ($u_{ij,2}(0)\leq 0.25e^{-3}$), the microscopic trend remains the same regardless of the gain (Fig. 5Ci, dark gray color). This is consistent with the idea that the network can use burst firing to first up-select newly encoded synaptic changes and later down-select them, a pattern compatible with homeostatic regulation. However, with intermediate connectivity, there is a range of gain values that promotes up-selection and thus consolidates memory during bursts. For very high connectivity, a weak gain will down-select, a mechanism compatible with synapse pruning that occurs during burst firing (6).

In summary, the two-factor synaptic plasticity rule can produce both up-selection and down-selection during burst firing. Up-selection reflects a consolidation-like regime in which the SNR increases during the burst state relative to the preceding tonic state, indicating that burst dynamics increase synaptic contrast by selectively strengthening informative connections relative to weaker ones, in the absence of replay. This behavior is consistent with the memorization task (“Two-factor synaptic plasticity promotes pattern consolidation during burst firing” section), where bursting increased the contrast of synaptic weight patterns (Fig. 3B). Conversely, down-selection corresponds to a decrease in SNR during burst firing, indicating reduced synaptic contrast (eg weakening of previously strengthened connections and/or compression of the separation between strong and weak weights). This down-selection is consistent with homeostatic renormalization or pruning-like downscaling during offline periods. Thus, by tuning the relative coupling between the two factors, the same framework can reproduce either selective stabilization (up-selection) or global downscaling (down-selection), linking our model to experimental and theoretical work on synaptic plasticity (46, 56, 57).

## Discussion

### Importance of burst firing in memory consolidation

Our model highlights a neuronal firing pattern that has been underexplored in synaptic plasticity research: collective burst firing within a network. Experimental studies typically focus on plasticity under highly controlled conditions, where spikes are precisely timed between presynaptic and postsynaptic neurons (58–61). In contrast, burst firing emerges from intrinsic neuronal and network mechanisms, and its complexity makes it harder to study experimentally. Computational models often sidestep this issue by representing rest as quiescence (41), yet our results suggest that the offline phase, when it involves structured burst activity, actively contributes to consolidation.

We show that including burst firing fundamentally changes how memories are stored. Neuronal activity naturally fluctuates between asynchronous (tonic-like) and synchronized (burst-like) regimes, depending on behavioral state and neuromodulation. In our model, bursts provide not just a pause between learning episodes but a distinct phase where synaptic weights are reorganized. Burst firing resets the primary factor, which, when coupled to the secondary factor, stabilizes prior learning. This suggests that combining tonic and burst states may be a general strategy for balancing flexibility in encoding with robustness in storage.

### Comparison with neo-Hebbian and cascade models

Many plasticity rules have been developed to incorporate additional mechanisms of learning and memory, from eligibility traces to cascade models. Our two-factor rule shares some of their goals but differs in key ways.

Eligibility-trace models compute a decaying variable from pre- and postsynaptic activity, which only influences plasticity when gated by a third factor such as reward or neuromodulation (62). In our framework, no extra variable is needed: bursting resets the primary factor, and the secondary factor captures stable processes. Consolidation is therefore tied to network state rather than to an external signal, and the effective weight is defined by the multiplicative interaction of the two variables.

Cascade models instead assign each synapse a hierarchy of hidden states with different stabilities, where plasticity events trigger transitions and memory lifetimes emerge from slow diffusion across states (33). In contrast, our rule uses only two variables and one coupling parameter without requiring multiple hidden states or stochastic transitions.

These frameworks should not be viewed as mutually exclusive. Future models may combine elements of cascade-like stabilization, eligibility traces, and state-dependent mechanisms such as ours, depending on the computational demands of the task (31).

### Implications for spiking neural networks and neuromorphic design

Beyond biological circuits, our findings also carry implications for artificial and neuromorphic networks. Most current spiking models rely exclusively on tonic spiking to represent and learn information. Here, alternating tonic and burst phases offer a new route for synaptic reorganization and consolidation (63, 64). The burst state acts as an “internal consolidation phase,” during which information is stabilized without replay of prior inputs. This provides a biologically inspired mechanism that could be integrated into neuromorphic systems to improve learning.

More generally, the fluctuating activity of biological circuits points to a useful design principle: networks should be trained not in a single spiking regime but with structured alternations between input-driven tonic activity and collective bursting modes. Our two-factor rule is particularly well suited to such applications. The effective weight is the product of two variables, with the secondary factor updated by a local derivative-based term. The coupling gain can be tuned as a single parameter, making the mechanism computationally lightweight and straightforward to implement in artificial networks or neuromorphic frameworks.

### Two-factor plasticity: simplicity, potential, and limitations

Finally, we place our approach in the broader landscape of plasticity rules, which span a wide spectrum—from short-term plasticity to structural plasticity, from detailed biophysical models of protein kinetics (65) to abstract spike-based correlation rules (21). Detailed biochemical models provide insight, but are often computationally expensive. In contrast, many abstract spike-based rules are formulated for tonic activity and are not designed to capture the functional consequences of burst firing.

An alternative framework for consolidation is STC (66, 67), in which potentiated synapses are locally tagged and stabilized through plasticity-related proteins. In computational models, STC is often implemented by the additive interaction of the primary and secondary factors (68, 69). While this additive formulation works under tonic firing, it fails during bursting: the burst firing drags all primary factors in the same direction, pulling the secondary factors with them.

The coupled two-factor rule proposed here offers a complementary, minimal alternative. Our formulation is directly inspired by recent work emphasizing that synaptic efficacy can be understood as the combined effect of multiple interacting subsynaptic processes and can be usefully represented through reduced-factor descriptions (24). Building on that perspective, we introduce a single coupling equation with one free parameter (the gain *η*), yet it captures a key functional effect: information encoded during tonic firing can be preserved while the primary factor is reset during burst firing. Importantly, we do not claim that this rule identifies the exact biochemical cascade engaged during burst epochs. Rather, we use two effective factors as a coarse-grained representation of many subsynaptic processes that likely interact during plasticity, including rapid changes in synaptic efficacy and slower stabilizing processes.

In this interpretation, the primary factor $u_{1}$ represents a flexible, tag-like component driven by correlated activity, while the secondary factor $u_{2}$ captures stabilizing processes recruited during burst epochs. The coupling between these factors through the rate of change should therefore be understood as a phenomenological proxy for coordinated pathway interactions, not as a one-to-one mapping to a single molecule or signaling branch (24). At a coarse-grained level, multiple interacting mechanisms (eg receptor trafficking, scaffold reorganization, phosphorylation-dependent state changes, and structural remodeling) could produce an effective coupling between stabilizing processes and the trajectory of the labile component. Candidate contributors include exocytotic regulators, activity-dependent trafficking programs (eg Arc), and kinase-dependent maintenance pathways (eg PKM*ζ*) (70, 71). In that sense, the model is biologically grounded but pathway-agnostic: it does not solve the molecular details, yet it makes a testable prediction about the *functional relationship* between labile synaptic changes and burst-gated consolidation.

This simplicity opens multiple avenues for extension. First, we assume that secondary plasticity is only active during bursts (η=0 in tonic, η>0 in bursts). In reality, stabilizing pathways may also occur during tonic activity; allowing a small negative *η* could let the secondary factors accumulate alongside primary potentiation, priming synapses for later consolidation. Second, we model *η* to be uniform across synapses, whereas biological coupling strength may vary with synaptic tags, cell type, or neuromodulatory context. Introducing heterogeneous *η* could support selective consolidation of salient memories. Third, we use a linear coupling to ${\dot{u}}_{1}$, but richer couplings (eg state-dependent, saturating, or history-dependent forms) may improve boundedness and stability. Exploring these extensions may bring the model closer to biological reality while preserving the phenomenological interpretability that makes the present formulation useful. Fourth, we restrict the model to a single burst-induced attractor. In principle, introducing additional inhibitory drives or neuromodulatory inputs could create multiple burst regimes and thus multiple attractors in synaptic dynamics, enabling distinct subsets of synapses to stabilize in different ways.

## Conclusion

We propose a simple two-factor synaptic plasticity rule in a conductance-based network that switches between tonic and burst firing. In this model, tonic firing encodes new external inputs, while burst firing stabilizes these changes through a secondary factor, without the need of replay. This mechanism supports memory retention and generalization, and shows that burst epochs can actively shape synaptic consolidation.

## Methods

### Neuron and network model

All neurons are modeled as single-compartment conductance-based neurons following Hodgkin and Huxley formalism (72). The membrane voltage *V* of a neuron evolves according to the equation described by (23, 25, 27):


$$C_{m}\dot{V}=-I_{\mathrm{leak}}-\sum_{\mathrm{ion}\in I}I_{\mathrm{ion}}-\sum_{\;p\in P}\sum_{\mathrm{syn}\in S}I_{\mathrm{syn},p}+I_{\mathrm{app}},$$

where $C_{m}$ represents the membrane capacitance, $I_{\mathrm{leak}}$ is the leak current, $I_{\mathrm{ion}}$ are the intrinsic ionic currents with I the set of all ionic channels, $I_{\mathrm{syn},p}$ are the synaptic currents with S the set of all synaptic neurotransmitter types and P the set of all presynaptic neurons, and $I_{\mathrm{app}}$ denotes the applied current. Details about the ionic and synaptic currents and their associated dynamics and parameters are provided in Ref. (25).

The neuronal network comprises an inhibitory neuron ($N_{\mathrm{inh}}$) projecting onto all excitatory neurons through ${\mathrm{GABA}}_{A}$ and ${\mathrm{GABA}}_{B}$ connections (refer to Fig. 1B). Excitatory neurons are interconnected via a feedforward AMPA synapse, where the presynaptic neurons ($N_{\mathrm{pre}}$) influence the postsynaptic neurons ($N_{\mathrm{post}}$) (25). The number of excitatory neurons varies across the different computational experiments. The excitatory synaptic current perceived by the postsynaptic neuron *i* from presynaptic neuron *j* is characterized by:


$$I_{\mathrm{AMPA},ij}=u_{ij,1}\cdot u_{ij,2}\cdot s_{\mathrm{AMPA},j}\cdot (V_{i}-E_{\mathrm{AMPA}}),$$

where $u_{ij,1}$ represents the primary factor, $u_{ij,2}$ is the secondary factor, and their product $u_{ij,1}\cdot u_{ij,2}$ defines the total synaptic weight. The variable $s_{\mathrm{AMPA},j}$ denotes the gating variable of the AMPA postsynaptic receptor (AMPAr), dynamically modulated by the presynaptic membrane voltage ($V_{\;j}$) and $E_{\mathrm{AMPA}}$ is the reversal potential of AMPAr. This model extends our previous representation of ${\bar{g}}_{\mathrm{AMPA},ij}$ as the maximal conductance of the AMPA receptors to accommodate primary and secondary synaptic plasticity, as outlined in Refs. (23, 25).

### Switch from tonic to burst firing

We follow the same method as in Ref. (25). The inhibitory neuron dynamically influences network activity. An external current applied to the inhibitory neuron ($I_{\mathrm{app},\mathrm{inh}}$) models the effects of a neuromodulatory signal. A depolarizing current induces tonic firing activity in this inhibitory neuron, causing all excitatory cells to remain at rest. A sufficiently large external depolarizing pulse can evoke action potentials in excitatory neurons. Conversely, a hyperpolarizing current applied to the inhibitory neuron switches the entire network into a synchronized collective bursting activity throughout the network (23, 73). In each computational experiment, the depolarizing current $I_{\mathrm{app},\mathrm{inh}}$ is equal to $3\;\mathrm{nA}/{\mathrm{cm}}^{2}$ for tonic state and $-1.2\;\mathrm{nA}/{\mathrm{cm}}^{2}$ for burst state.

### External applied pulse train current

We follow the same method as in Ref. (25). In either tonic firing, quiescent states, or noisy states, each excitatory neuron *i* is triggered by an applied current ($I_{\mathrm{app},i}$) to make the neuron fire at a nominal frequency $f_{0}$. To generate this input-driven tonic activity, a neuron receives a pulse train current, where each pulse lasts 3ms and has an amplitude that is independently sampled from a uniform distribution on an interval between 50 and $60\;\mathrm{nA}/{\mathrm{cm}}^{2}$. The interpulse intervals (ie the time between two successive pulses) are independently sampled from a Normal distribution with a mean equal to $1/f_{0}$ and a standard deviation equal to $0.1/f_{0}$.

In burst firing states, each excitatory neuron *i* undergoes a noisy baseline such as the applied current ($I_{\mathrm{app},i}$) randomly fluctuating between the interval 0 and $1\;\mathrm{nA}/{\mathrm{cm}}^{2}$.

### Homogeneous and heterogeneous network

We follow the same method as in Ref. (25). We define a *homogeneous* network (or circuit), where each neuron has the same intrinsic ion channel maximal conductances ${\bar{g}}_{\mathrm{ion}}={\bar{g}}_{\mathrm{ion}}^{*}$ (numerical values are provided in Ref. (25) and in Supplementary material). We define a *heterogeneous* network (or circuit), where the maximal conductance ${\bar{g}}_{\mathrm{ion}}$ for each neuron is randomly chosen within a ±10 interval around its nominal value ${\bar{g}}_{\mathrm{ion}}^{*}$, such as ${\bar{g}}_{\mathrm{ion}}={\bar{g}}_{\mathrm{ion}}^{*}(1+\epsilon )$ with ϵ∼Unif(−0.1,0.1).

### Local field potential

We follow the same method as in Ref. (25). The local field potential (LFP) measures the average behavior of interacting neurons. It reflects the collective excitatory synaptic activity received by the postsynaptic neuron population. The overall synaptic activity is measured by the mean of the individual synaptic currents:


$$\mathrm{LFP}(t)=-\frac{1}{M}\sum_{\;j=1}^{M}\sum_{i=1}^{N}I_{\mathrm{AMPA},ij}(t),$$

where *M* is the number of postsynaptic neurons and *N* is the number of presynaptic neurons (23, 27).

### Synaptic plasticity

#### Primary synaptic plasticity

The change in the primary factor $u_{ij,1}$ between a presynaptic neuron *j* and a postsynaptic neuron *i* is governed by the calcium-based model proposed by (34):


(3) $$\begin{array}{rl} \tau_{u_{1}}{\dot{u}}_{ij,1} & =-u_{ij,1}(1-u_{ij,1})(u_{ij,1}^{*}-u_{ij,1})\\ & \;-\gamma_{d}u_{ij,1}\Theta ([{\mathrm{Ca}}^{2+}{]}_{ij}-\theta_{d})\\ & \;+\gamma_{p}(1-u_{ij,1})\Theta ([{\mathrm{Ca}}^{2+}{]}_{ij}-\theta_{p}). \end{array}$$

Here, $\tau_{u_{1}}$ represents the time constant, $u_{1}^{*}$ defines the stable state (equal to 0.5), $\gamma_{p}$ is the potentiation rate, $\gamma_{d}$ is the depression rate, $\theta_{p}$ is the potentiation threshold, and $\theta_{d}$ is the depression threshold. The function Θ(⋅) is the Heaviside function, which returns 1 if $[{\mathrm{Ca}}^{2+}{]}_{ij}>\theta_{p}$ and 0 otherwise.

The change in primary factor depends on the calcium concentration $[{\mathrm{Ca}}^{2+}{]}_{ij}$, which is the sum of the calcium caused by the activity of the presynaptic neuron *j* and the activity of the postsynaptic neuron *i*. A pre- or postsynaptic spike translates into a calcium exponential decay. For details about the calcium dynamics, see Supplementary material, Calcium dynamics. The calcium-based rule defined by (34) implements a soft-bound, where the perceived potentiation rate $\gamma_{p}(1-u_{ij,1})$ is smaller for high $u_{ij,1}$ than for low $u_{ij,1}$. The same reasoning applies to the depression rate $\gamma_{d}$. Detailed parameter values are provided in Supplementary material, Computational experiments.

#### Secondary synaptic plasticity

The secondary factor $u_{ij,2}$ between the presynaptic neuron *j* and the postsynaptic neuron *i* evolves according to Eq. 2. The coupling gain *η* is set to 1/500 in Fig. 2 and 1/400 in Fig. 3, and varies from 1/1,000 to 1/10 in Fig. 5.

The threshold $\theta_{\mathrm{thres}}$ was not assigned a single fixed value but adjusted to ensure clear separation between tonic and burst regimes in each network instantiation. In practice, tonic firing emerges for $I_{\mathrm{app},\mathrm{inh}}≳0$, while collective bursting is robust for $I_{\mathrm{app},\mathrm{inh}}≲-1$. Exact values are not critical, reflecting the high degree of degeneracy typical of biophysical networks.

#### Signal-to-noise ratio

The signal-to-noise ratio calculated in Fig. 5 is calculated following the method proposed by (46):


(4) $$\mathrm{SNR}=\frac{\max_{i,j}(u_{ij,1}\cdot u_{ij,2})}{{\mathrm{mean}}_{i,j}(u_{ij,1}\cdot u_{ij,2})}.$$

### Computational experiments

Details about the computational experiments are available in Supplementary material.

## Supplementary Material

pgag213_Supplementary_Data

## Data Availability

All original data in this work were generated using the Julia programming language (74). Analyses were performed in Matlab. The code files are freely available at https://github.com/KJacquerie/Two-Factor-Plasticity. Any additional information can be requested from the lead contact (kathleen.jacquerie@gmail.com).

## Preprints

A preprint of this article is published at doi.org/10.1101/2025.04.12.648539.
