# Supplementary material for: Two-factor synaptic plasticity enables memory consolidation during neuronal burst firing
Source: PNAS Nexus. 2026 Jun 12;5(7):pgag213. doi: 10.1093/pnasnexus/pgag213 (PMC13323795; doi:10.1093/pnasnexus/pgag213)
Supplement: pgag213_Supplementary_Data [file pgag213_supplementary_data.pdf]

Supplementary Material

Calcium dynamics

We consider the linear calcium dynamics suggested by (Graupner and Brunel, 2012; Graupner et al., 2016). The presynaptic and postsynaptic spike contributions add linearly. Indeed, calcium enters from NMDA receptors and voltage-dependent calcium channels. Instead of describing the whole calcium, the phenomenological effect on the calcium variation is considered. At each pre or postsynaptic spike (respectively named as $t_{j,k}$ and $t_{i,k}$), the calcium immediately rises and then exponentially decays characterized by a calcium decay time constant equal to τ_{Ca}:

$$\frac{dc_j}{dt} = -\frac{c_j}{\tau_{\text{Ca}}} + C_{\text{pre}} \sum_{k \in \mathcal{T}_j} \delta(t - t_{j,k} - D),$$
$$\frac{dc_i}{dt} = -\frac{c_i}{\tau_{\text{Ca}}} + C_{\text{post}} \sum_{k \in \mathcal{T}_i} \delta(t - t_{i,k}),$$

where C_{pre} and C_{post} are the presynaptically and postsynaptically evoked calcium amplitudes. The parameter D is a time-delay between the presynaptic spike and the corresponding postsynaptic calcium transient occurrence accounts for the slow rise time of the NMDAr-mediated calcium influx (Graupner and Brunel, 2012; Graupner et al., 2016).

The total calcium amplitude $[\text{Ca}^{2+}]_{ij}(t)$ driving the synaptic change is given by:

$$[\text{Ca}^{2+}]_{ij}(t) = c_j(t) + c_i(t).$$

The time-evolution for several pre- and postsynaptic spiking activity is written such as (Graupner et al., 2016):

$$[\text{Ca}^{2+}]_{ij}(t) = \sum_{k \in \mathcal{T}_j} C_{\text{pre}} \exp\left(\frac{t - t_{j,k} - D}{\tau_{\text{Ca}}}\right) + \sum_{k \in \mathcal{T}_i} C_{\text{post}} \exp\left(\frac{t - t_{i,k}}{\tau_{\text{Ca}}}\right).$$

The resting calcium concentration is set to zero. The calcium concentrations are dimensionless. Both simplification is acknowledged because the synaptic rules are adapted in accordance. If a resting calcium concentration is wanted, the thresholds of potentiation and depression will be adapted. This notation follows the original paper notation.

Numerical values

The parameters associated with the primary synaptic plasticity are given in (Graupner and Brunel, 2012). $\tau_{\text{Ca}} = 22.6936\,\text{ms}$, $C_{\text{pre}} = 0.56$, $C_{\text{post}} = 1.24$, $D = 4.60\,\text{ms}$, $\tau_{u_1} = 346.3615 \times 10^3\,\text{ms}$, $\gamma_{\text{p}} = 725.085 \times 1.1$ (Tonic), $\gamma_{\text{p}} = 725.085 \times 0.95$ (Burst), $\gamma_{\text{d}} = 331.909$, $\theta_{\text{p}} = 1.3$, $\theta_{\text{d}} = 1$, $u_1^* = 0.5$. The potentiation rate γ_p is slightly scaled up during tonic firing to induce stronger potentiation compared to the initial model, and it is reduced by 5 % during burst firing to place the reset at a lower value compared to the initial model.

The parameters used in each simulation are $N = 50, M = 50, T_{\text{state}} = 20\,\text{s}, N_{\text{state}} = 4$ for Figure 2, $N = 484, M = 10, T_{\text{state}} = 15\,\text{s}, N_{\text{state}} = 62$ for Figures 3-4, $N = 100, M = 1, T_{\text{state}} = 20\,\text{s}, N_{\text{state}} = 10$ for Figure 5. Some parameters remain constant in the different simulations: $I_{\text{app,inh}}(\text{Tonic}) = 3\,\text{nA/cm}^2$, $I_{\text{app,inh}}$ (Burst) $= -1.2\,\text{nA/cm}^2$, $u_1(0) = 0.5$, $u_2(0) = 0.001$, $\eta = 1/500$ in Figure 2, $1/400$ in Figure 3. In Figure 5, $u_2(0)$ varies from 0.0001 to 0.01 and η varies from $1/1000$ to $1/10$. To constrain the secondary weight, a maximum upper bound is present ($u_{2,\text{max}}$) - this bound is never reached in the different computational experiments. The minimum and maximum values of w are, respectively, $0.0265e^{-3}$, 0.00342 for Figure 3B, $0.168e^{-3}$, $0.733e^{-3}$ for Figure 3C, and $0.176e^{-3}$, $0.706e^{-3}$ for Figures 5A-B.

Supplementary Material related to Figure 1

Figure 1C illustrates the activity in the feedforward network, comprising 50 presynaptic neurons connected to 50 postsynaptic neurons. The network is heterogeneous. The network is in tonic firing mode during 1.5 s. Each excitatory neuron receives a train of current pulses with nominal frequencies f_0 randomly sampled from a uniform distribution between 0.1 and 50 Hz. Then, the network switches to synchronized collective bursting during 2.5 s.

Supplementary Material related to Figure 2

Figures 2A-D show results from a small heterogeneous circuit comprising one inhibitory neuron providing GABAergic input to four excitatory neurons, where two presynaptic excitatory neurons project onto two postsynaptic excitatory neurons.

The computational experiments include four configurations: alternating tonic and burst states (Figures 2A-C, tonic states interleaved with quiescent periods (Figure 2C), or continuous tonic states (Figure 2D). In Figure 2A, primary and secondary plasticity are active, while in Figure 2B both the secondary plasticity is blocked ($\dot{u}_2 = 0$).

During tonic states, excitatory neurons are stimulated with pulse trains at fixed nominal frequencies. In the first tonic state, the two presynaptic neurons fire at 60 Hz and 1 Hz, and the postsynaptic neurons at 35 Hz and 5 Hz. In the second tonic state, presynaptic neurons fire at 65 Hz and 1 Hz, while postsynaptic neurons fire at 30 Hz and 5 Hz. These values were selected to illustrate potentiation and depression during tonic activity. .

During burst states, the inhibitory neuron is hyperpolarized, producing collective burst firing. In the quiescent condition, all neurons are driven by low-frequency pulse trains randomly selected from 0.1 Hz, 0.5 Hz, or 1 Hz.

In the additional tonic condition (Figure 2D), firing frequencies are randomly drawn from the set $\{0.5, 1, 5, 10, 40, 50\}$ Hz.

Supplementary Material related to Figure 3

Figure 3A illustrates the simulation protocol for a learning task interleaved with periods of burst firing. The whole task lasts 6030 s (*i.e.*, 100 min and 30 s). The protocol is divided into 21 sequences (Roman numerals). In each sequence (I-XX), the network sequentially learns the digits 0 to 9. Each sequence lasts a total of 300 s and is composed of 10 blocks of tonic and burst firing. Each block lasts 30 s with a tonic or a burst firing state of 15 s each. The final sequence (XXI) lasts 30 s and consists of only one block. A cropped version of the MNIST dataset (LeCun et al., 1998) with 22×22 pixels is used. The initial dataset of 28×28 pixels has 3 pixels cropped on each side. The training set is composed of 20 samples of each digit pulled in the training set composed of 80 samples.

The network consists of 484 (22×22) presynaptic neurons representing individual image pixels and 10 postsynaptic neurons representing the digit classes (0 to 9). One inhibitory neuron projects onto all these neurons. The network is homogeneous to eliminate confounding factors while studying variations in the synaptic plasticity rule (parameters are available in (Jacquerie et al., 2025)).

A learning state in the network corresponds to tonic activity (dark blue). Excitatory neurons are initially at resting membrane potential. Presynaptic neurons are activated by a train of current pulses with a nominal frequency reflecting the pixel color. The MNIST images are binarized such that a pixel with an intensity greater than 0.5 is considered a white pixel otherwise it is black. A white pixel is encoded by a neuron spiking at 45 Hz and a black pixel at 0.01 Hz. The postsynaptic neuron corresponding to the presented digit class spikes at 45 Hz, while the others spike at 0.0001 Hz. Each sequence is composed of 10 blocks. The learning states are sequentially organized from digit 0 to digit 9, with one sample digit randomly chosen from the MNIST dataset for each digit class.

An off state corresponds to either collective bursting activity (light blue) or quiescent activity (lavender) in the network. For the collective bursting activity, all excitatory neurons do not receive

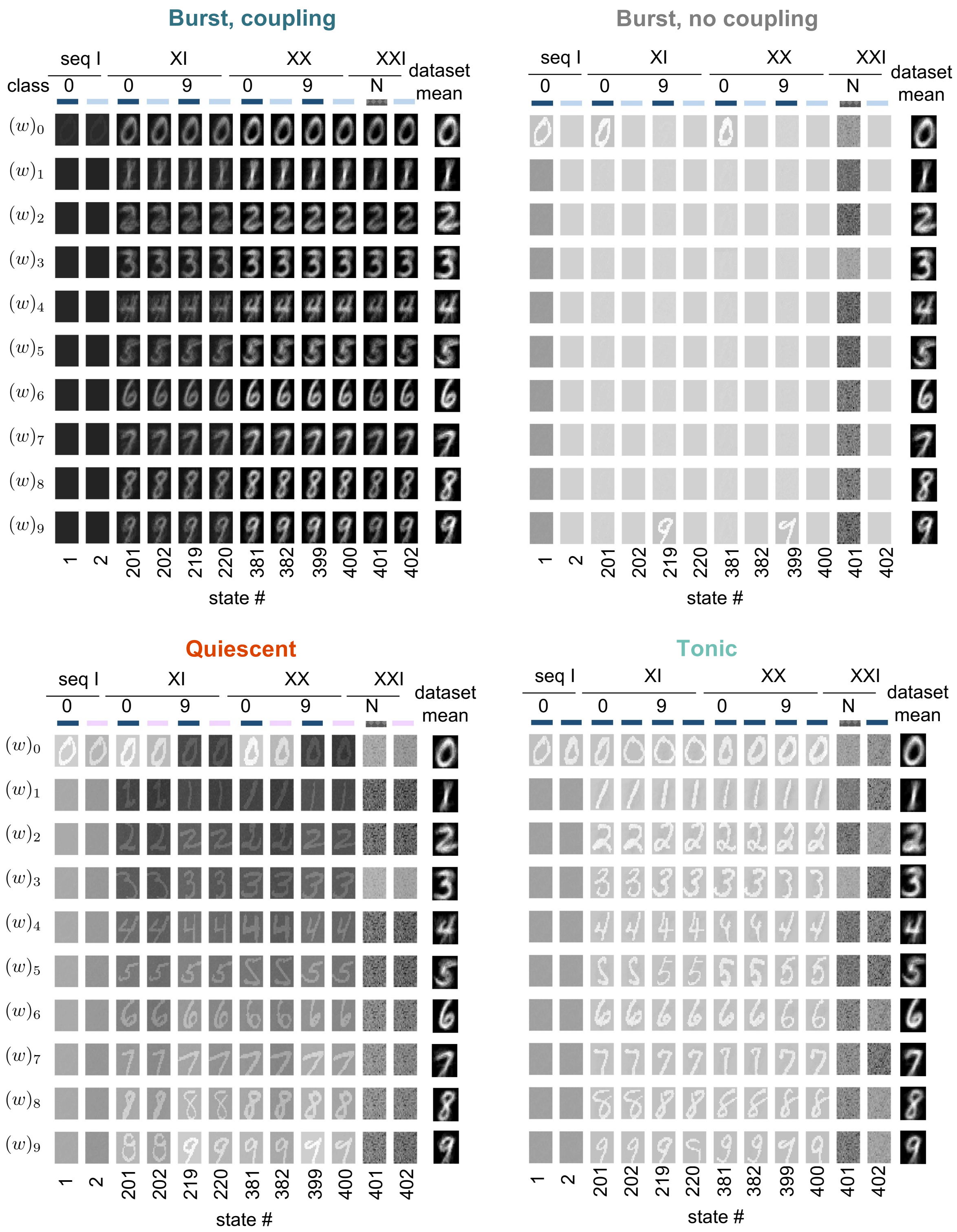

Figure S1: Evolution of the weight matrices for the 10 digits at different states, associated with Figure 3 for the different models (Burst, coupling; Burst, no coupling; Quiescent; Tonic), depicts the protocol that interleaves different firing activities depending on the model color coded by tonic (dark blue), burst firing (light blue), noise (for N, in gray) and quiescent (lavender). (seq means sequence).

any input-driven pulse but only undergo baseline noise. For the quiescent activity, all neurons receive a pulse train with nominal frequencies independently sampled from a Normal distribution with a mean equal to 1 Hz and a standard deviation equal to 0.1 Hz.

A noisy state (gray) occurs during sequence XXI for 15 s. In this state, all excitatory neurons

receive a pulse train with nominal frequencies independently sampled from a Normal distribution with a mean equal to 15 Hz and a standard deviation equal to 5 Hz.

Figures 3B-E illustrate the weight matrix of each digit. It corresponds to the total synaptic weights of the 484 presynaptic neurons onto the postsynaptic neuron associated with the considered digit, reshaped in a 22×22 grid matrix. The color of each pixel is proportional to the synaptic weight matrix $(w)_i$ acquired at the end of each sequence. For visualization purposes, synaptic weights are normalized so that the normalized synaptic weight values throughout the whole simulation span the interval $(0, 1)$, that is,

$$(\widetilde{w})_i = \frac{(w)_i - \min_{i,j,t}(w_{ij}(t))}{\max_{i,j,t}(w_{ij}(t)) - \min_{i,j,t}(w_{ij}(t))},$$

where the maximum and minimum values are obtained by comparing the values across all pairs of presynaptic and postsynaptic neurons at the end of each state throughout the whole simulation. The weight matrices are shown for the states 1, 2 (in sequence I), 201, 202, 239, 240 (in sequence XI), 399, 400 (in sequence XX), 401, and 402 (in sequence XXI) for the digit 0 and 9. Other digits can be seen in Figure S1.

Figure 3B alternates between tonic and burst firing with the two-factor plasticity rule ($\eta = 1/400$). Figure 3C blocks the secondary synaptic plasticity with a coupling gain of 0 in burst firing too. Figure 3D replace the bursting state by a quiescent state. Figure 3E is identical to Figure 3D, except that the bursting state (light blue) is replaced by an additional learning state for the same digit, using a different randomly chosen sample of the same digit. The weight matrices are obtained in the same manner, and the normalization is achieved by selecting the maximum and minimum values in this simulation. They differ from those in Figure 3B.

Supplementary Material related to Figure 4

We compare the effect of normalization on training and testing consolidation percentage. Figures S2 A-B show results for unnormalized weights, while Figures S2 C-D display normalized weights. Testing involves unseen samples of digits 0 to 3. Models without secondary plasticity, where only primary weights fluctuate, perform poorly, while our "Burst, coupling" model excels in the training set and can generalize its learning on the testing set. Normalization improves the performance of other models but still lags behind our approach.

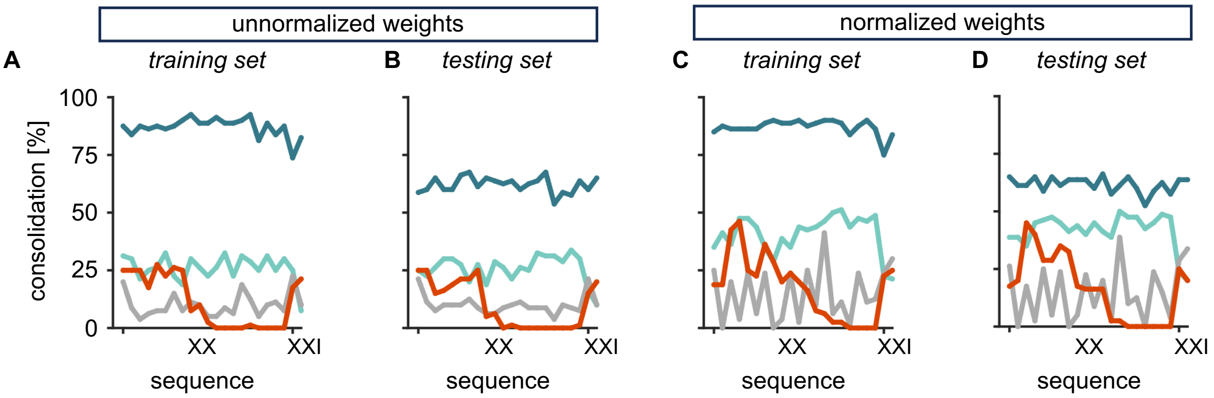

Figure S2: Comparison of weight normalization on consolidation percentage for training and testing datasets. Consolidation percentage was obtained for the final two sequences (XX-XXI) without weight normalization**(A, B.)**, compared to with normalized weights **(C, D)**. Consolidation percentage is compared between the training dataset **(A, C)** and the testing dataset **(B, D)**.

Supplementary Material related to Figure 5

Figure 5A illustrates a heterogeneous network comprising one inhibitory neuron connected to 101 excitatory neurons, where 100 presynaptic neurons synapse onto 1 postsynaptic neuron. The stimulation protocol consists of 10 states interleaving tonic firing and burst firing, each lasting 15 s.

For each tonic state, the first five neurons (dark purple) receive an applied current that consists of a train of pulses with a frequency independently sampled for each neuron from a uniform distribution between 73 and 76 Hz (the frequencies are resampled at the beginning of each tonic state). The other 95 presynaptic neurons (lilac) receive a pulse train with a frequency independently sampled for each neuron from a uniform distribution between 0.1 and 5 Hz. The postsynaptic neurons receive a pulse train at 25 Hz (purple). These values are selected to demonstrate strong and weak correlations, although other ranges of values also yield results. The pulse train is constructed as previously described. The raster plot and the LFP are computed for 4 s during the first state transition (Figure 5B).

Figure 5C (top) highlights the trend followed by the SNR for different values of the coupling gain (η) and the initial value of the secondary factor ($u_{ij,2}(t = 0)$). Figure 5Ci to iv are obtained for the following values: (i) $\eta = 1/200$, $u_{ij,2}(t = 0) = 0.001$; (ii) $\eta = 1/400$, $u_{ij,2}(t = 0) = 0.005$; (iii) $\eta = 1/90$, $u_{ij,2}(t = 0) = 0.0075$; (iv) $\eta = 1/50$, $u_{ij,2}(t = 0) = 0.0025$.

Different primary plasticity rules can shape different learning strategies for overlapping patterns

We examine how the choice of the primary plasticity rule influences memory consolidation in the two-factor synapse model. Although the burst-induced attractor emerges robustly in multiple rules (Jacquerie et al., 2025), the way information is consolidated can differ substantially, particularly when patterns share overlapping features (*i.e.*, when two images of different categories share common pixels).

We compare two versions of the calcium-based model that governs the primary synaptic plasticity: the model proposed by Graupner and Brunel (2012) and the one introduced by Graupner et al. (2016). The 2012 model posits that at low calcium concentrations, the primary weight follows a cubic function with two stable states, while the 2016 model suggests that the primary weight remains constant under low calcium concentrations. In both cases, the secondary plasticity rule remains the same.

To test their impact, we design a simplified recognition task with 16 presynaptic neurons encoding 4×4 pixel images and 2 postsynaptic neurons ($N = 16, M = 2$, Figure S3A). Each pattern consists of two bars, presented either as non-overlapping (Figure S3B) or overlapping (Figure S3C). Training alternates between tonic and burst states (Figure S3D). During each tonic state, one bar (4 pixels) of the pattern is presented and the subsequent burst state consolidates it before the second bar is introduced. This reflects a scenario where partial features of a class are learned incrementally. We study how the different circuit consolidate the different bars and build the total pattern by studying the weight matrices and the correlation, as it captures how closely synaptic weights resemble to the patterns.

For non-overlapping patterns, both the 2012 and 2016 models allow successful representations of the patterns in the weight matrices. Correlation traces increase steadily across training sequences (Figures S3 Ei–ii), and the final weight matrices match the input patterns (Figures S3 Eiii–iv). To quantify selectivity, we compute the contrast between the average synaptic strength of pixels forming the pattern and those outside it. In the 2012 model, contrast is 17% for pattern 1 and 20% for pattern 2, whereas in the 2016 model it rises to 39% for both. Thus, both models support recognition, but the 2016 model yields sharper representations.

For overlapping patterns, the models diverge. With the 2012 rule, both patterns are maintained, as shown by steadily increasing correlations (Figure S3 Fi) and final contrasts of 19% and 21%. In contrast, with the 2016 rule, the network fails to reinforce shared pixels: the non-overlapping parts are encoded, but overlapping pixels fade (Figures S3Fii–iv). The resulting contrast drops to 10% for both patterns, and the correlation of one pattern increases only at the expense of the other. The evolution of the weight matrices throughout the protocol in both cases is shown in Figure S4.

These differences arise because the trajectory of the primary weight u_1 directly shapes how the secondary weight u_2 evolves during bursts. Variations between the 2012 and 2016 calcium-based rules—different parameter values, plasticity constants, or nonlinear terms such as the cubic component in the 2012 formulation—alter the dynamics of u_1, which in turn modifies consolidation through u_2. Although it is difficult to isolate a single factor behind these divergent outcomes, the important point is that our two-factor framework is adaptable: it can be combined with different primary rules, and whichever pathway governs u_1 will determine how u_2 evolves. In this way, the same mechanism can operate across diverse configurations, producing distinct strategies that favor either integration or separation depending on the underlying primary dynamics.

The 2012 model favors integration, preserving both patterns even when they overlap, while the 2016 model favors separation, encoding only distinct features and discarding shared ones. This resembles how humans sometimes extrapolate from partial cues to reconstruct a full object, yet in other contexts fail to recognize it from fragments alone. In biological circuits, different pathways likely underlie primary plasticity, creating a diversity of consolidation outcomes depending on input structure and context. Our two-factor rule does not interfere with these primary pathways but builds on them, suggesting that biological diversity in plasticity rules may allow circuits to flexibly balance generalization and discrimination according to task demands.

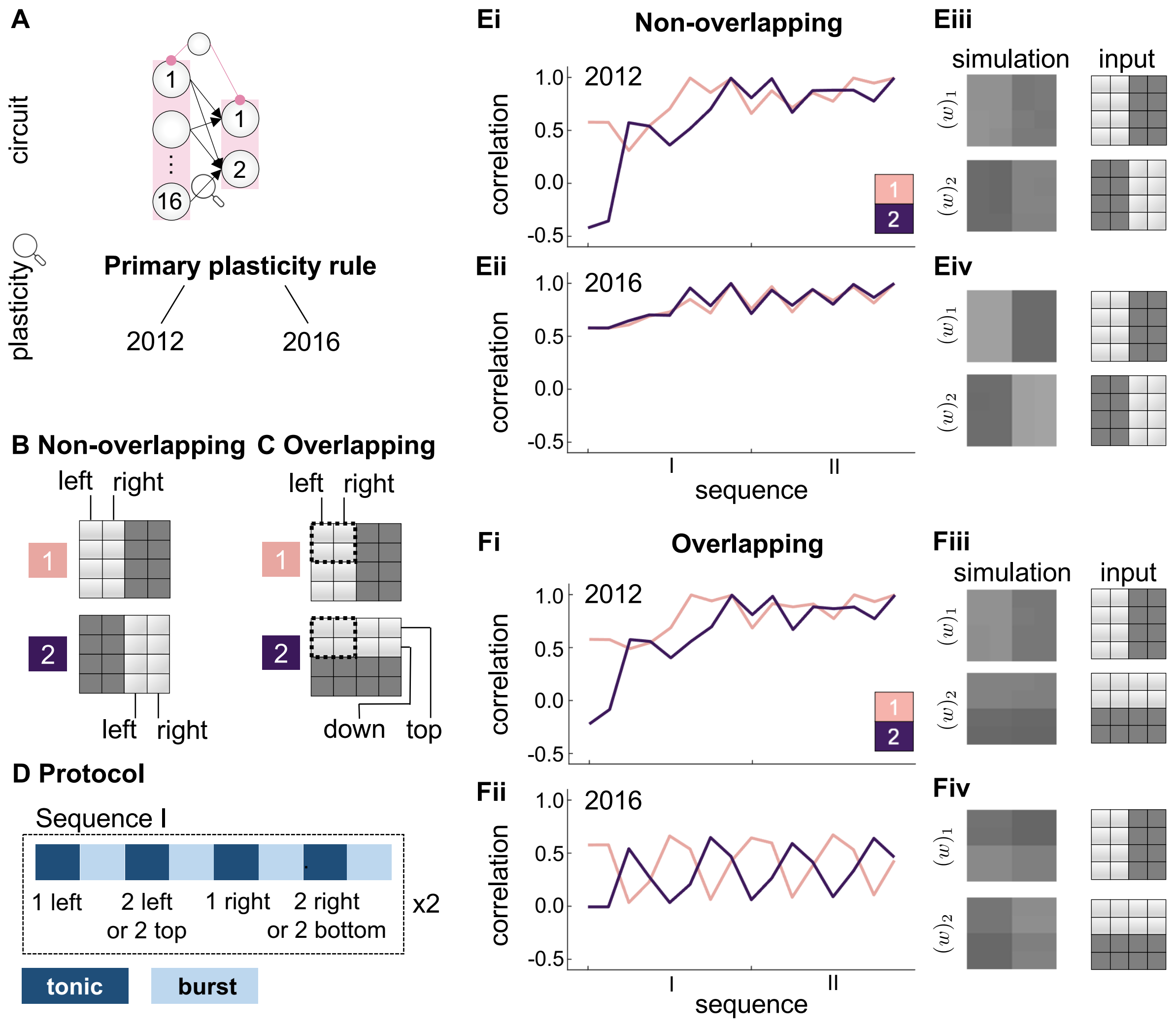

Figure S3: Different primary plasticity rules produce distinct strategies for consolidating overlapping patterns. A. Circuit: 16 presynaptic neurons encode 4×4 pixel inputs and project to 2 postsynaptic neurons. Excitatory synapses follow either the 2012 calcium-based rule (Graupner and Brunel, 2012) or the 2016 rule (Graupner et al., 2016), combined with secondary plasticity. **B.** The task involves categorizing either non-overlapping patterns (#1, #2) or **C.** overlapping patterns (#1, #2) on a 4x4 grid image. Each pattern is composed of two bars (left, right, or top, down). Shared pixels shown with dashed outline **D.** Training protocol: each sequence alternates tonic (input-driven learning of one bar) and burst states. Each pattern consists of two bars presented sequentially. **E.** Non-overlapping patterns. **(Ei–ii)** Evolution of correlations for the two patterns under the 2012 (Ei) and 2016 (Eii) rules. **(Eiii–iv)** Final weight matrices show successful encoding for both rules, with stronger contrast in the 2016 model. **F.** Overlapping patterns. **(Fi–ii)** Correlations under the 2012 and 2016 rules. The 2012 model consolidates both patterns despite overlap, while the 2016 model suppresses shared pixels. **(Fiii–iv)** Final weight matrices confirm distinct learning strategies.

Methods

Figure S3A illustrates a homogeneous network comprising one inhibitory neuron connected to 18 excitatory neurons, where 16 presynaptic neurons synapse onto 2 postsynaptic neurons. Synapses undergo synaptic plasticity where the primary weight is either driven by the primary plasticity fitted in the calcium-based rule of (Graupner and Brunel, 2012) (2012 model) or (Graupner et al., 2016) (2016 model). The secondary plasticity is active across both scenarios.

The network is trained to learn two patterns, each corresponding to 8 active pixels out of the 16-pixel grid. The simulation protocol consists of two sequences of 4 tonic firing states interleaved with four burst firing states. During each tonic state, only 4 out of the 8 pixels are learned. It corresponds to creating samples for the same class. To achieve this, the corresponding input neurons are activated

by a pulse train at 40 Hz, while the output neuron associated with the pattern class is triggered by a pulse train at 55 Hz. Inactive input pixels receive a pulse train at 0.01 Hz, and the second output neuron receives a pulse train at 0.0001 Hz. We alternate between learning one part of a pattern and, in the subsequent tonic firing state, one part of the other pattern. Correlation is computed as detailed in the methods section for Figure 4.

To compute the percentage difference, μ_{in} is defined as the mean of the pixels within the pattern, and μ_{out} as the mean of the pixels outside the pattern. In the non-overlapping case, for the first pattern, μ_{in} is the mean of the first two columns, and μ_{out} is the mean of the third and fourth columns. For the second pattern, these are reversed. In the overlapping case, μ_{in} and μ_{out} are calculated similarly, except for the second pattern, where μ_{in} is the mean of the first two rows, and μ_{out} is the mean of the third and fourth rows. The percentage difference is defined as $(\mu_{\text{in}} - \mu_{\text{out}})/((\mu_{\text{in}} + \mu_{\text{out}})/2)$.

For Figure S3, the network parameters are $N = 16, M = 2, T_{\text{state}} = 15\,\text{s}, N_{\text{state}} = 16$, $\eta = 1/200$ and $u_2(0)$ equals 0.02.

In Figure S3, the calcium-based model is modified according to the version of (Graupner et al., 2016). The model is similar except that the stable state is removed and the fitted plasticity parameters are adapted:

$$\tau_w \dot{u}_{ij,1} = \gamma_{\text{p}}(1 - u_{ij,1})\Theta([\text{Ca}^{2+}]_{ij} - \theta_{\text{p}}) - \gamma_{\text{d}} u_{ij,1}\Theta([\text{Ca}^{2+}]_{ij} - \theta_{\text{d}}). \tag{5}$$

Figure S4 depicts the evolution of the weight matrices associated with Figure S3 across successive tonic and burst firing states. In Figure S4A, the weight matrices are compared for non-overlapping conditions between the 2012 model and the 2016 model. Similarly, Figure S4B compares the weight matrices under overlapping conditions between the 2012 model and the 2016 model.

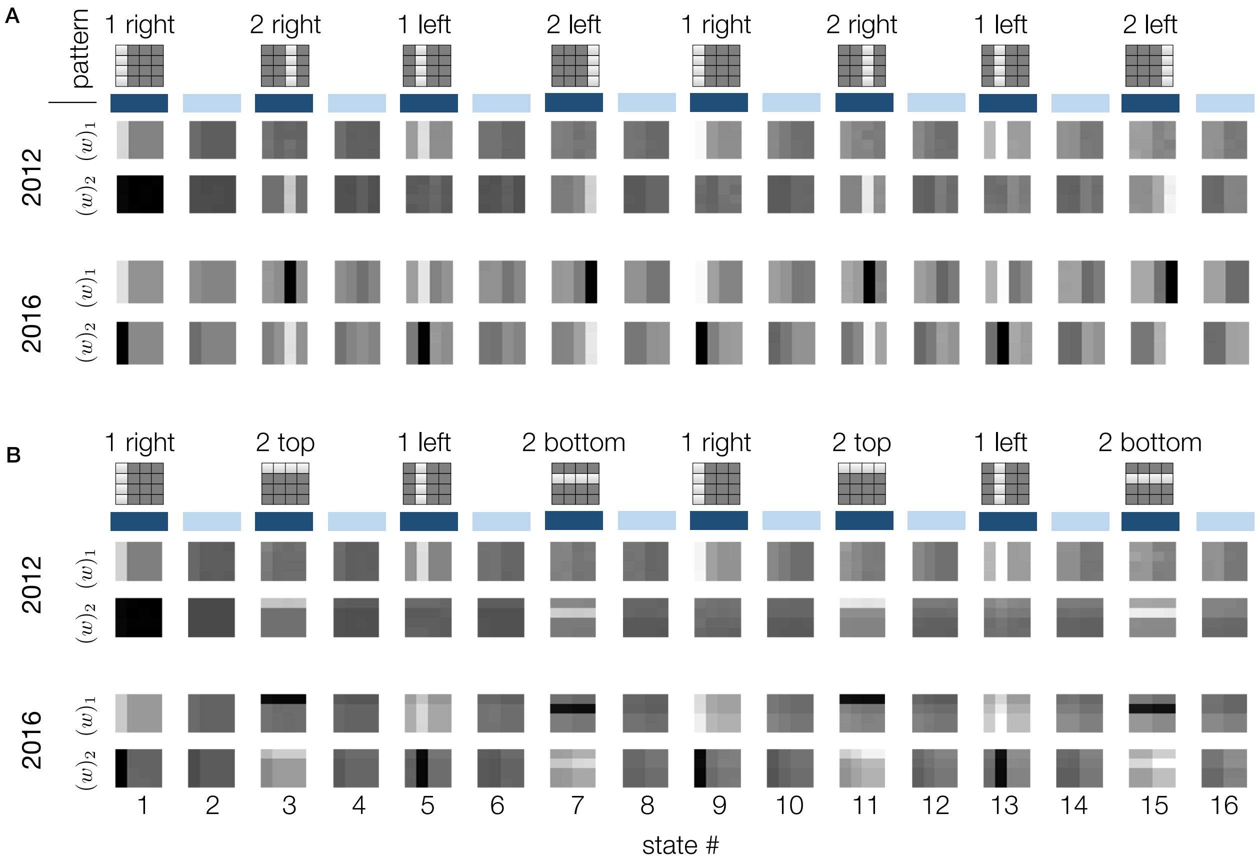

Figure S4: Evolution of the weight matrices for the 2 patterns at different states, associated with Figure S3, **A.** for non-overlapping patterns or **B.** overlapping patterns
